# Neuroprotective Effect of Luteolin-7-O-Glucoside against 6-OHDA-Induced Damage in Undifferentiated and RA-Differentiated SH-SY5Y Cells

**DOI:** 10.3390/ijms23062914

**Published:** 2022-03-08

**Authors:** Stephanie Cristine Hepp Rehfeldt, Joana Silva, Celso Alves, Susete Pinteus, Rui Pedrosa, Stefan Laufer, Márcia Inês Goettert

**Affiliations:** 1 Cell Culture Laboratory, Graduate Program in Biotechnology, University of Vale do Taquari (Univates), Lajeado 95914-014, RS, Brazil; srehfeldt@universo.univates.br; 2 Marine and Environmental Sciences Centre (MARE), Polytechnic of Leiria, 2520-630 Peniche, Portugal; joana.m.silva@ipleiria.pt (J.S.); celso.alves@ipleiria.pt (C.A.); susete.pinteus@ipleiria.pt (S.P.); 3 MARE—Marine and Environmental Sciences Centre, ESTM, Polytechnic of Leiria, 2520-614 Peniche, Portugal; rui.pedrosa@ipleiria.pt; 4 Department of Pharmaceutical and Medicinal Chemistry, Institute of Pharmacy, Eberhard Karls Universität Tübingen, D 72076 Tubingen, Germany; stefan.laufer@uni-tuebingen.de; 5 Tübingen Center for Academic Drug Discovery (TüCAD2), D 72076 Tubingen, Germany

**Keywords:** 6-hydroxydopamine, apoptosis, mitochondrial membrane potential, neurodegenerative diseases, oxidative stress, cell culture techniques, neurodegenerative disorders, neuroprotective effect, biological products

## Abstract

Luteolin is one of the most common flavonoids present in edible plants and its potential benefits to the central nervous system include decrease of microglia activation, neuronal damage and high antioxidant properties. The aim of this research was to evaluate the neuroprotective, antioxidant and anti-inflammatory activities of luteolin-7-O-glucoside (Lut7). Undifferentiated and retinoic acid (RA)-differentiated SH-SY5Y cells were pretreated with Lut7 and incubated with 6-hydroxydopamine (6-OHDA). Cytotoxic and neuroprotective effects were determined by MTT assay. Antioxidant capacity was determined by DPPH, FRAP, and ORAC assays. ROS production, mitochondrial membrane potential (ΔΨm), Caspase–3 activity, acetylcholinesterase inhibition (AChEI) and nuclear damage were also determined in SH-SY5Y cells. TNF-α, IL-6 and IL-10 release were evaluated in LPS-induced RAW264.7 cells by ELISA. In undifferentiated SH-SY5Y cells, Lut7 increased cell viability after 24 h, while in RA-differentiated SH-SY5Y cells, Lut7 increased cell viability after 24 and 48 h. Lut7 showed a high antioxidant activity when compared with synthetic antioxidants. In undifferentiated cells, Lut7 prevented mitochondrial membrane depolarization induced by 6-OHDA treatment, decreased Caspase-3 and AChE activity, and inhibited nuclear condensation and fragmentation. In LPS-stimulated RAW264.7 cells, Lut7 treatment reduced TNF-α levels and increased IL-10 levels after 3 and 24 h, respectively. In summary, the results suggest that Lut7 has neuroprotective effects, thus, further studies should be considered to validate its pharmacological potential in more complex models, aiming the treatment of neurodegenerative diseases.

## 1. Introduction

Neurodegenerative diseases (ND) are comprised of distinct and heterogeneous disorders characterized by the progressive and selective loss of neurons. Usually, the prevalence and symptoms worsening are intimately related with age, and as the global population gets older, the need for new ND therapeutic alternatives and deeper knowledge of their pathophysiology is urgent. The World Health Organization (WHO) estimates that by 2040, ND such as Alzheimer’s disease (AD), and other types of dementia, or conditions that compromises motor function like Parkinson’s disease (PD) or amyotrophic lateral sclerosis (ALS), will be the second most prevalent cause of death, after cardiovascular diseases [1]. However, due to the complexity and heterogeneity of NDs, most of the synthetic drugs evaluated in in vitro models and/or clinical trials end up failing [2], and thus further efforts must be conducted to develop an efficient disease-modifying treatment.

Luteolin, a phytochemical belonging to the flavone class of polyphenols, is one of the most common flavonoids present in edible plants. Its potential benefits to the CNS include the decrease of microglia activation and neuronal protection [3,4,5]. However, the glycosylated form of luteolin, known as cyranoside or luteolin-7-O-glucoside (Lut7) (PubChem ID 5280637), was reported as a selective JNK3 inhibitor, five times more selective than luteolin [6], which plays a key role in neurodegenerative diseases [7,8,9,10,11], suggesting that patients with neurodegenerative diseases might benefit from a natural or bio-inspired product-based therapy [12,13,14,15,16].

The effects of herbal extracts containing Lut7 as a majority compound are extensively explored in the literature. However, it is not possible to confirm that those reported effects are a result of the Lut7 activity or if other components are mediating the observed effects. Therefore, the aim of this study was to evaluate the antioxidant and anti-inflammatory activities of Lut7, as well as its neuroprotective effects in an in vitro human neurodegenerative model (SH-SY5Y cells induced with 6-OHDA) in both undifferentiated and differentiated cells.

## 2. Materials and Methods

### 2.1. Cell Lines and Reagents

Dulbecco’s modified Eagle medium (DMEM) (D5523), F12 (N6760), heat-inactivated fetal bovine serum (FBS) (F4135), 6-hydroxydopamine hydrobromide (6-OHDA) (162957), 3-[4,5-dimethylthiazol-2]-2,5 diphenyltetrazolium bromide (MTT) (M5655), Penicillin (P3032), streptomycin (S9137), lipopolysaccharide (LPS) (from *Escherichia coli*, O111:B4, L2630), trypsin-EDTA (T4049), 2′,7′-dichlorofluorescin diacetate (DCFDA) (D6883), Caspase-3 Activity Fluorimetric kit (CASP3F), 2,4,6-Tris(2-pyridyl-s-triazine (TPTZ) (T125), FCCP, and oligomycin A were purchased from Sigma-Aldrich™ (St. Louis, MO, USA). DMEM (1200-058) used to culture RAW264.7 cell line and enzyme-linked immunosorbent assay (ELISA) kits for TNF-α, IL-6, and IL-10 were acquired from Gibco^®^, Invitrogen Life Science Technologies (Grand Island, NY, USA). All-trans-retinoic acid (ATRA) (SC200898) was purchased from Santa Cruz Biotechnology, (Dallas, TX, USA). Spectrophotometer analysis was performed using a SpectraMax^®^ i3 microplate reader (Molecular Devices, San Jose, CA, USA). 5,5,6,6-tetrachloro-1,1,3,3-tetraethylbenzimidazolylcarbocyanine iodide (JC-1) staining was acquired from Molecular Probes (Eugene, OR, USA) and 4,6-diamidino-2-phenylindole (DAPI) staining was obtained from Applichem (Darmstadt, Germany). Photographs for the DAPI probe were taken with a fluorescence microscope Zeiss, model Axio Vert. A1, (Oberkochen, Germany). Lut7 (26-S) was obtained from Extrasynthese (Genay, Cedex, France). SH-SY5Y (ATCC^®^ CRL-2266™) and RAW264.7 cell line (ATCC^®^ TIB-71™) were acquired from American Type Culture Collection (ATCC). MAPK inhibitors (SP600125 and SB203580) were synthesized by Prof. Dr. Stefan Laufer research laboratory with a high purity grade (≥95%).

### 2.2. Cell Culture Methods

#### 2.2.1. SH-SY5Y Cell Line 

Undifferentiated human SH-SY5Y neuroblastoma cells were cultured in DMEM mixed with F12 (1:1) and supplemented with 10% (*v/v*) FBS and 1% streptomycin/penicillin under controlled conditions in a 95% humidified atmosphere, at 37 °C and 5% CO_2_. Culture medium was replaced every two days until the cells reached confluence 4–5 days after the initial seeding. For subculture, SH-SY5Y cells were dissociated with trypsin-EDTA, split into a 1:3 ratio. Cells were grown to 80% confluence before treatment. Culture conditions were performed according to ATCC recommendations.

#### 2.2.2. SH-SY5Y Differentiation Protocol 

The differentiation of SH-SY5Y cells was carried out in two steps. Firstly, SH-SY5Y cells were cultivated in media with 1% FBS supplemented with 10 µM of all-trans retinoic acid (ATRA) for 4 days. At the 5th day, the cell culture medium was replaced by fresh medium and cells were cultivated for 6 days.

#### 2.2.3. RAW264.7 Cell Line 

RAW264.7 cells were cultured in DMEM supplemented with 10% FBS (*v*/*v*) and 1% streptomycin/penicillin. The medium was replaced every 2 to 3 days. Sub-culturing was carried out with a cell scraper at a 1:4 split ratio. All procedures were performed according with ATCC recommendations. 

### 2.3. Determination of Cell Viability and Neuroprotection Potential by MTT Assay

#### 2.3.1. Evaluation of Lut7 Cytotoxicity

Cell viability was assessed using the colorimetric MTT assay [17] with slight modifications as described by Rehfeldt et al. [11]. SH-SY5Y (differentiated and undifferentiated) and RAW264.7 cells were seeded in 96-well dishes and left overnight for proper attachment. Cells were exposed to different concentrations (10–0.1 μM) of Lut7 over 24 or 48 h. MTT reagent was then added to each well at a final concentration of 5 mg/mL and the plate was placed in a humidified incubator at 37 °C with 5% CO_2_ during 3 h. Formazan salts were dissolved in DMSO and the colorimetric determination of MTT reduction was estimated at 570 nm wavelength using the SpectraMax^®^ i3 microplate reader. Untreated cells were used as control and considered as 100% viable. 

#### 2.3.2. Neuroprotection Potential

Neuroprotection effect was assessed using the colorimetric MTT assay with slight adaptations [11,17,18]. To investigate the neuroprotective potential of the compound, SH-SY5Y (differentiated and undifferentiated) cells were seeded at a density of 2 × 10^4^ per well in a 96-well dish and cultivated overnight. Cells were then treated with different concentrations (1 or 0.1 μM) of Lut7 over 30 min before adding 6-OHDA (100 μM) stabilized with 0.02% of ascorbic acid to avoid its auto-oxidation. Cells’ medium was then removed following 24 or 48 h of treatment and MTT (5 mg/mL) solution was added and the cells were incubated for 3 h. Following the MTT removal, DMSO was used to dissolve the formazan salts and the absorbance read at 570 nm.

### 2.4. Determination of Antioxidant Activity

The antioxidant activity of Lut7 was evaluated by the means of different methodologies, namely (a) 2,2-diphenyl-1-picrylhydrazyl (DPPH) radical scavenging ability [19]; (b) oxygen radical absorbance capacity (ORAC) [20]; and (c) ferric reducing antioxidant power (FRAP) assays [21] with slight adaptations [15]. Butylated hydroxytoluene (BHT) was used as a positive control for antioxidant activity.

### 2.5. Mechanisms of Cell Recovery after 6-OHDA-Induced Damage 

#### 2.5.1. Mitochondrial Membrane Potential (MMP) Assay

Cells were seeded in 96-well plates and left overnight in the incubator. SH-SY5Y cells were then treated with 6-OHDA (100 μM) for 6 h, in the absence or presence of Lut7 (0.1 or 1 μM). After exposure, cells were washed with PBS 1× and incubated with JC-1 at 37 °C for 30 min. Then, the reagent was gently removed, and cells were washed with PBS 1×. 100 μL/well PBS 1× was added and the fluorescence was measured at 490/595 nm (red fluorescence) and 490/530 nm (green fluorescence) of excitation and emission wavelengths, respectively [11,22]. FCCP (2.5 µM) plus oligomycin A (1 µg/mL) conjugate solution was used as positive control. Mitochondrial membrane potential was estimated by measuring the fluorescence of free JC-1 monomers (green) and JC-1 aggregates in mitochondria (red) and the results were expressed as the ratio of the monomers/aggregates of JC-1 in percentage of control. 

#### 2.5.2. ROS Production

The levels of reactive oxygen species (ROS) were determined using the 5-(and-6)-carboxy-2′, 7′-dichlorodihydrofluorescein diacetate (carboxy-H2DCFDA) probe as previously described [11,22] with slight modifications. Briefly, SH-SY5Y cells were treated with Lut7 at different concentrations (0.1 or 1 μM) and 6-OHDA (100 μM) over 6 h. Following treatment, cells were washed with PBS (1×) and20 μM carboxyH2DCFDA solution, previously dissolved in serum-free medium, and cells were incubated for 1 h at 37 °C. The fluorescence was read at 527 nm and 495 nm wavelength of emission and excitation, respectively. 

#### 2.5.3. Caspase 3 Activity

The enzyme was assessed using the Caspase-3 Activity Fluorometric kit, according to manufacturer’s instructions. SH-SY5Y cells were cultured in 6-well plates and treated with 6-OHDA (100 μM) for 6 h in the presence or absence of Lut7 (0.1 or 1 μM). Caspase-3 activity was calculated from the slope of the linear phase of the fluorescence resulting from the rhodamine 110 accumulation and expressed in arbitrary fluorescence units per mg protein per minute (∆ fluorescence (a.u.)/mg protein/min) [22].

#### 2.5.4. DAPI Staining

The nucleic condensation and/or fragmentation was determined by 4,6-diamidino-2-phenylindole (DAPI) staining. SH-SY5Y cells were cultured in 6-well plates and treated with 6-OHDA (100 μM) for 24 h in the presence or absence of Lut7 (0.1 or 1 μM). The cells were fixed in paraformaldehyde (4%) over 30 min. After this time, the solution was removed, and cells were incubated in Triton X-100 (0.1%) over 30 min. Then, Triton X-100 was removed, followed by the addition of DAPI (1 μg/mL) solution. After a 30 min reaction, DAPI was removed, and 1 mL PBS (pH 7.4) was added to each well. Then, the cells were observed using an Axio Vert. A1 fluorescence microscope (Zeiss, Oberkochen, Germany) [22].

#### 2.5.5. AChE Activity

AChE activity was measured spectrophotometrically in a 96-well microplate according to a modified Ellman assay [23,24]. SH-SY5Y cells were cultured in 6-well plates overnight and treated with 6-OHDA (100 μM) for 6 h, in the absence or presence of Lut7 (0.1 or 1 μM). Then, cells were trypsinized with PBS (1×) + 0.1% Triton X-100. The supernatant was transferred to a 96-well microplate and incubated with DTNB (0.5 mM) and acetylcholine (Ach; 1 mM). The absorbance was measured at λ = 414 nm.

### 2.6. Cytokine Levels in RAW264.7 Cell Line

RAW264.7 cells were seeded at a density of 5 × 10^5^ cells per well in a 24-dish plate. After adherence time, cells were pretreated for 1 h before LPS (1 μg/mL) treatment. The supernatant was collected at different times as described by Rehfeldt and co-workers [11]. To evaluate TNF-α release, samples of supernatant were collected after 3 and 24 h of treatment. For IL-6 analysis, samples were collected after 12 h of treatment, and for IL-10 after 48 h of treatment. All samples were frozen at −80 °C until further analysis. ELISA was performed according to the manufacturer’s instructions. The absorbances were measured at 450 nm and 570 nm using a spectrophotometer (SpectraMax^®^ i3). Values of 570 nm were subtracted from those of 450 nm to remove background interference. TNF-α, IL-6 and IL-10 standard curves were used to quantify the release from each cytokine by the cells.

### 2.7. Data and Statistical Analysis

The statistical analysis was performed on GraphPad Prism 6.0 software (GraphPad software, San Diego, CA, USA) using ANOVA. The results are expressed in mean ± standard error of the mean (SEM). Differences were considered significant at level of 0.001 (*** *p* < 0.001), 0.01 (** *p* < 0.01), and 0.05 (* *p* < 0.05). At least three independent experiments carried out in triplicate were performed.

## 3. Results

### 3.1. Cytotoxic and Neuroprotective Effect of Lut7 in Undifferentiated and RA-Differentiated SH-SY5Y Cells

**Figure 1:**
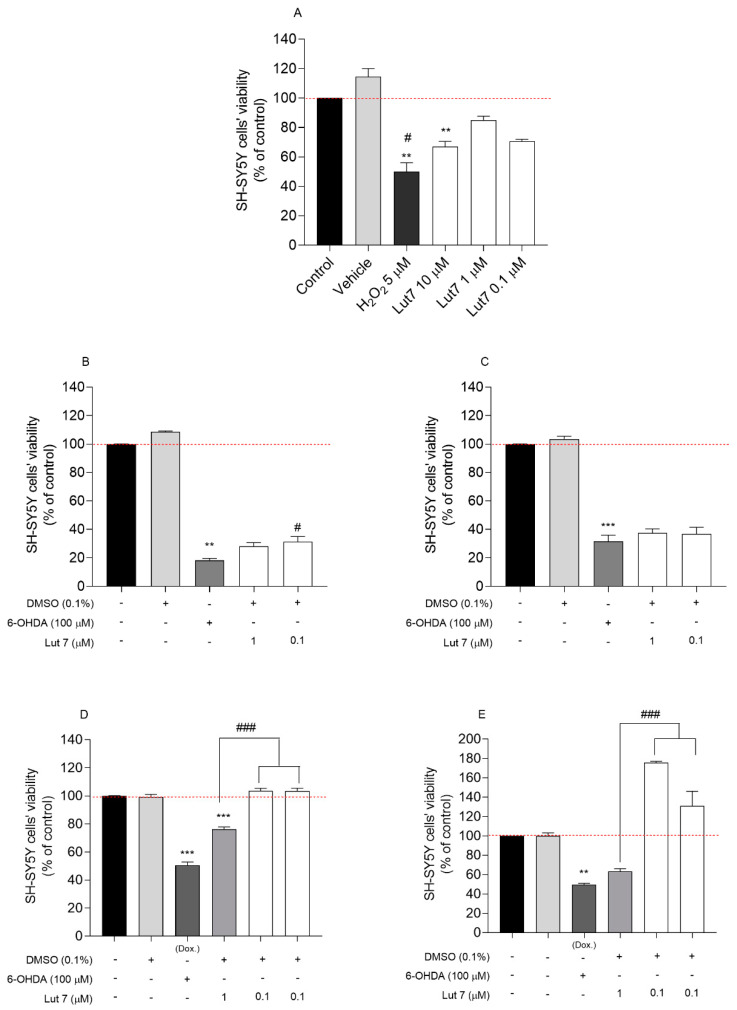
Cytotoxicity induced by Luteolin-7-O-glucoside (Lut7) in SH-SY5Y cells after 24 h of treatment (**A**); Neuroprotective effects of Lut7 against 6-OHDA induced neurotoxicity on undifferentiated SH-SY5Y cells: 24 h incubation (**B**); 48 h incubation (**C**). Neuroprotective effects of Lut7 against 6-OHDA induced neurotoxicity on RA-differentiated SH-SY5Y cells: 24 h incubation (**D**); 48 h incubation (**E**). The values in each column represent the mean ± SEM of at least three independent experiments carried out in triplicate. Statistical calculations were performed with ANOVA via the Tukey post hoc test and significant differences were considered for *** *p* < 0.001; ** *p* < 0.01 (vs. control); ### *p* < 0.001 and # *p* < 0.05 (vs. 6-OHDA). Doxorubicin was used as positive control; DMSO 0.1% was used as vehicle. Negative control (untreated cells) was considered to be 100% viable.

The first set of experiments examined the impact of Lut7 on cell viability. The results shown in Figure 1A indicate that 10 µM Lut7 reduced SH-SY5Y cells viability by 33% (*p* < 0.05). At 1 µM and 0.1 µM, the compound did not significantly decrease the cells’ viability, thus, these concentrations were selected for the neuroprotective assays.

The capacity of undifferentiated SH-SY5Y cells to recover from 6-OHDA stimuli was evaluated. Cells were pretreated with Lut7 at sub-toxic concentrations over 1 h before 6-OHDA treatment. The exposition of SH-SY5Y cells to 6-OHDA (100 µM) significantly reduced the cell viability after 24 h when compared to the untreated cells. However, when cells were treated with 0.1 µM Lut7, there was a 13% increase in cell viability when compared with 6-OHDA treatment (Figure 1B). However, this effect was not observed after 48 h (Figure 1C). 

After differentiation, cells start to upregulate genes involved with antioxidant defense. This modified profile of gene expression reflects directly in the capacity of cells to recover from the oxidative stress caused by 6-OHDA. To confirm this higher resistance, one positive control with doxorubicin (10 μM) was included. The mechanism of action of doxorubicin is achieved by specifically blocking the activity of the enzyme topoisomerase II, which is involved in DNA replication during mitosis, and does not interfere in oxidative stress and, therefore, does not impact the damage caused by 6-OHDA. In this sense, SH-SY5Y cells were differentiated 10 days before neuroprotective assay. RA-differentiated SH-SY5Y cells pretreated with Lut7 at 1 and 0.1 μM increased cell viability in 27.4 and 27.1%, respectively (*p* < 0.001) (Figure 1D). On the other hand, after 48 h, RA-differentiated SH-SY5Y cells pretreated with 1 and 0.1 μM Lut7 increased cell viability in 112% (*p* < 0.001) and 67.5% (*p* < 0.001), respectively, when compared with 6-OHDA treatment (Figure 1E).

### 3.2. Lut7 Antioxidant Activity

**Figure 2:**
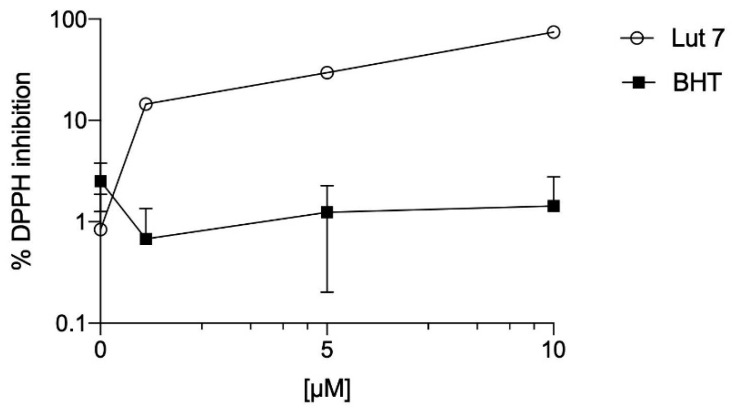
DPPH scavenging ability of Lut 7 and BHT—dose response analysis.

The antioxidant activity of Lut7 was evaluated by three different chemical methods: (a) DPPH assay to determine the capacity of Lut7 scavenging potential; (b) FRAP to determine the Lut7 capacity to reduce ferric ions; (c) ORAC method to evaluate the presence of antioxidant molecules with the ability to neutralize the peroxyl radicals. The results are presented in Table 1 and Figure 2.

It was observed that Lut7 presented the highest potential of scavenging DPPH radical with an EC_50_ value of 6.80 µM when compared to the standard BHT (EC_50_ > 100 µM). In the ORAC method, Lut7 showed the highest antioxidant activity with 8804.19 ± 409.99 µmol of Trolox/g compound, when compared with BHT (143.70 ± 23.36 µmol of Trolox/g compound). Lut7 was also effective in reducing ferric ions (19,570.78 ± 291.48 µM FeSO_4_/g of compound) when compared with the synthetic antioxidant.

### 3.3. Lut7 Effects on Cellular Hallmarks Associated with ND

**Figure 3:**
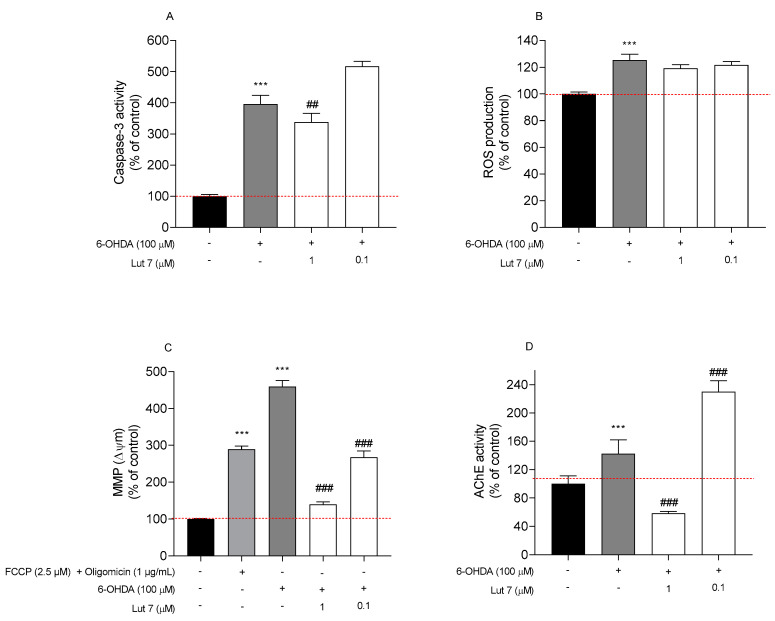
Effect of Luteolin-7-O-glucoside (Lut7) on SH-SY5Y cells after exposition to 6-OHDA (100 µM) over 6 h. (**A**) Effect on Caspase-3 activity (Δ Fluorescence (U.A)/mg of protein/min); (**B**) ROS production; (**C**) MMP (ratio of monomers/aggregates of JC-1); and (**D**) AChE activity (nmol/min/mg of protein). The values in each column represent the mean ± standard error of the mean (SEM) of at least 3 independent experiments carried out in triplicate. Statistical calculations were performed with ANOVA via the Tukey post hoc test and significant differences were considered for *** *p*< 0.001 (vs. control); ### *p* < 0.001 and ## *p* < 0.01 (vs. 6-OHDA). Negative control (untreated cells) was considered to be 100% viable and is represented by the red dashed line. DMSO 0.1% was used as a vehicle.

Hallmarks of apoptotic cell death include the activation of caspases, the disruption of mitochondrial membrane potential and DNA fragmentation. These same events are also present during neurodegenerative diseases. To verify if the neuroprotective effect demonstrated by Lut7 on undifferentiated SH-SY5Y cells was associated with these hallmarks, different in vitro assays on cells treated with neurotoxin 6-OHDA in the absence or presence of Lut7 were carried out (Figure 3).

Firstly, the Caspase-3 activity was measured to understand if the Lut7 had capability to prevent the cell death mediated by apoptosis when exposed to the 6-OHDA neurotoxin. At 1 µM, Lut7 was able to decrease Caspase-3 activity by 57.6% (Figure 3A).

Secondly, the ability of Lut7 to prevent a condition of oxidative stress was evaluated through ROS quantification. After exposing SH-SY5Y cells to 6-OHDA (100 µM, 6 h) a two-fold increase in ROS levels was verified when compared to vehicle. However, Lut7 was not able to decrease ROS levels induced by 6-OHDA treatment (Figure 3B). 

Thirdly, the MMP was determined to evaluate mitochondrial dysfunction and to understand if the neuroprotective effects of Lut7 were mediated by biological events that usually take place on mitochondria. Treatment with 6-OHDA at 100 µM for 6 h increased depolarization of SH-SY5Y cells mitochondrial membrane potential in 359.2% when compared to vehicle. On the other hand, the treatment with Lut7 (1 µM) exhibited a protective effect against cell depolarization induced by 6-OHDA treatment in 320% (139.2% vs. 459.2% of 6-OHDA). At 0.1 µM, Lut7 showed a similar effect decreasing the monomers/aggregates ratio of JC-1) in 192% (Figure 3C).

Considering the mechanism of action of many FDA-approved drugs to treat AD, the AChE activity was evaluated after Lut7 treatment. It is well documented that the AChE inhibition decreases the breakdown and promotes the accumulation of ACh, therefore, compensating the loss of functional cholinergic neurons and alleviating cognitive symptoms of AD. The results presented here showed that 6-OHDA treatment increased AChE activity in 43.47%. On the other hand, cells treated with 1 µM Lut7 reduced AChE activity in 77.49 ± 8.63% when compared with 6-OHDA treatment (Figure 3D). Cells pre-treated with 0.1 µM Lut7 followed by 6-OHDA exposure showed approximately 1-fold increase in AChE activity (242.67 ± 8.9%) when compared to the neurotoxin.

**Figure 4:**
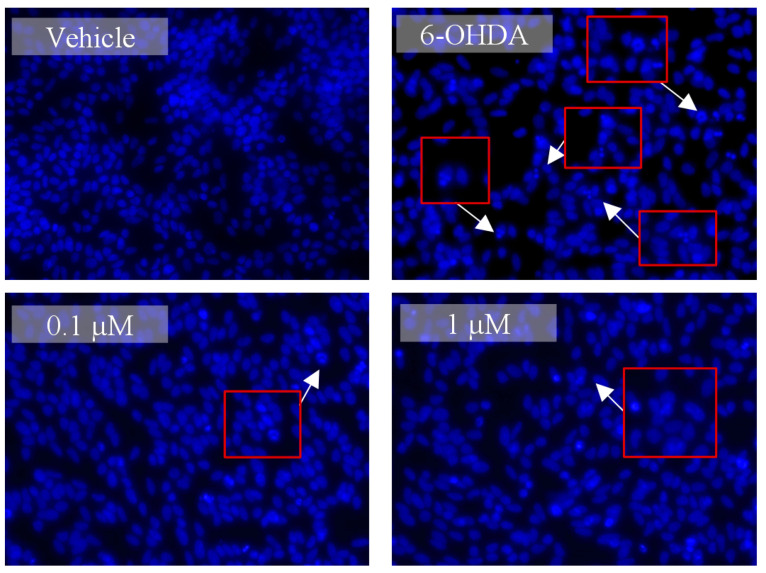
Nuclear morphology of SH-SY5Y cells stained with DAPI probe. SH-SY5Y cell stained with DAPI showing the anti-apoptotic effect of the Lut7 (0.1 or 1 µM) against neurotoxicity mediated by 6-OHDA (100 µM; 24 h). Arrows show nuclear abnormalities (fragmentation pattern), which is an indicator of apoptosis. Red Boxes represent the amplified zone where is visible nuclear changes.

Finally, to understand if Lut7 had the ability to prevent the DNA fragmentation induced by 6-OHDA treatment, the integrity of SH-SY5Y DNA was observed following DAPI staining (Figure 4).

The exposition of SH-SY5Y cells to 100 µM 6-OHDA over 24 h led to nuclear condensation and fragmentation, which are characteristic features of apoptosis. However, it was possible to verify that Lut7 decrease the occurrence of those events induced by 6-OHDA.

### 3.4. Cytokines Levels on LPS-Induced RAW264.7 Cells Treated with Lut7

**Figure 5:**
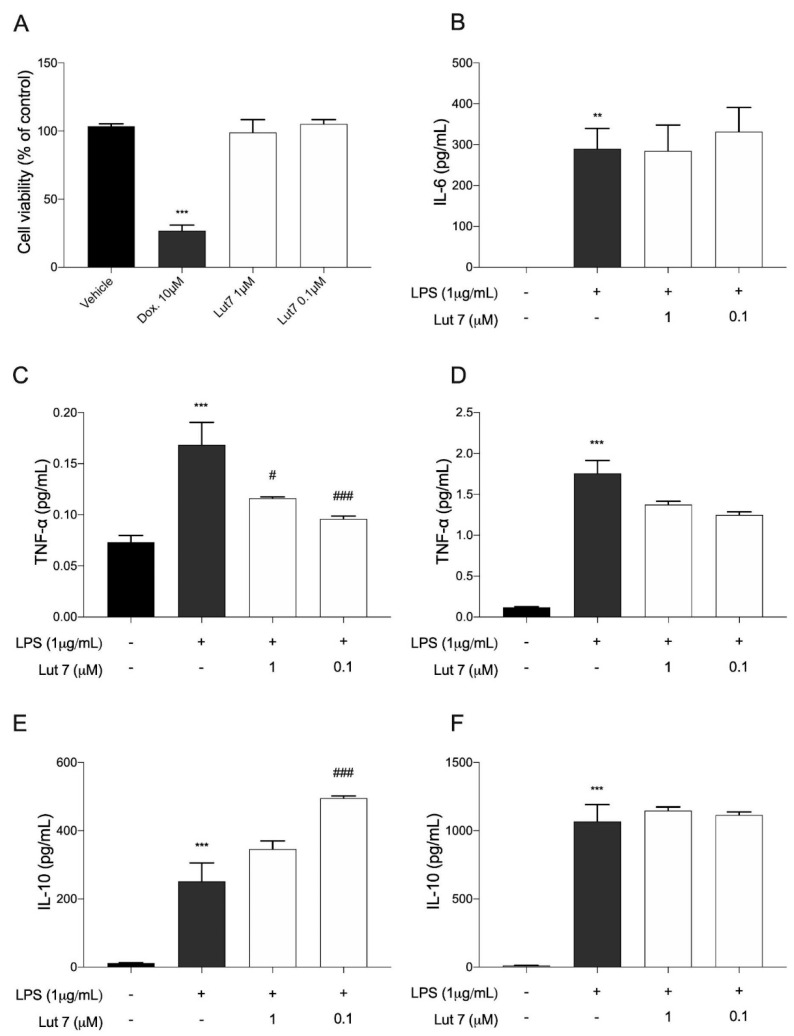
(**A**) Cytotoxicity of luteolin-7-O-glucoside (Lut7) on RAW264.7 cells viability after treatment for 24 h; (**B**) IL-6 levels were determined after 12 h of treatment; (**C**) TNF-α levels were determined after 3 h and (**D**) 24 h of treatment. IL-10 levels were estimated (**E**) after 24 h; and (**F**) 48 h of treatment. The values represent the mean ± SEM of at least three experiments carried out in triplicate. ** *p* < 0.01; *** *p* < 0.001; (vs. control); ^###^ *p* < 0.001 and ^#^ *p* < 0.05 (vs. LPS). Doxorubicin was used as positive control; DMSO 0.1% was used as vehicle.

Cytokines play a crucial role in the inflammatory response. Before evaluating the effects of Lut7 treatment over cytokine release, its cytotoxic effects on the RAW267.4 cells viability were evaluated (Figure 5). 

Since at 10 µM, Lut7 reduced SH-SY5Y cells’ viability, the cytotoxic effect of Lut7 in RAW264.7 cells was only tested at 1 and 0.1 µM. It was verified that at 0.1 and 1 μM, Lut7 did not induce cytotoxicity in RAW 264.7 cells (Figure 5A), and thus, these concentrations were used in the following assays. It was verified that, at both concentrations, Lut7 did not stimulate IL-6 release (Figure 5B). On the other hand, after 3 h, cells pretreated with 1 μM and 0.1 Lut7 decreased TNF-α levels in 30.97% and in 43.02%, respectively (Figure 5C). Lut7 treatment showed no effect over TNF-α levels after 24 h (Figure 5D). Concerning IL-10 release, which is delayed, the supernatant was collected after 24 and 48 h of treatment. Only after 24 h, Lut7 at 0.1 μM was able to stimulate the IL-10 release in 96.9% (Figure 5E). However, these effects were not maintained over 48 h (Figure 5F).

## 4. Discussion

Pre-clinical studies reported that luteolin, a flavonoid present in many fruits and vegetables, has anti-inflammatory and antioxidative properties [25,26,27,28]. However, the impact on neuroprotection using the RA-differentiated SH-SY5Y cell line has been poorly explored. Despite both differentiated and undifferentiated SH-SY5Y cells being used in experiments as suitable in vivo models of NDs, authors suggest that cells should be differentiated since undifferentiated cells are prevenient from a metastatic tumor and continuously undergo division, making it difficult to predict the effect of protective agents against neurotoxins. Indeed, differences in gene expression profiles, antioxidant capacity, synthesis of neurotransmitters and other phenotypic aspects have been observed between differentiated and undifferentiated cells [29,30,31]. Additionally, authors evaluated the enzymatic activity of AChE and choline acetyltransferase (ChAT) (cholinergic markers) in both differentiated and undifferentiated SH-SY5Y cells and demonstrated that cells show a different pattern of AChE activity when treated with RA [32]. Those findings suggest that the SH-SY5Y cell line may respond to the 6-OHDA stimuli differently, depending on whether they are differentiated or not.

In undifferentiated SH-SY5Y cells, our findings did not reveal a significant neuroprotection effect. These results are in line with a previous study performed with Lut7, in which the compound also did not show significant differences in SH-SY5Y cells’ viability when treated with an amyloidogenic molecule [33]. On the other hand, on RA-differentiated cells, Lut7 showed a marked protective effect against 6-OHDA-induced damage, probably due to its increased ability to promote the expression of genes related to antioxidant defenses. This is consistent with previous studies where RA-induced differentiation of SH-SY5Y cells has been related with resistance to oxidants, possibly due to modulation of ROS production and oxidative stress responses [34,35,36,37]. This result ties well with a previous study wherein intensified oxidative phosphorylation in differentiated SH-SY5Y was observed [38]. 

The results herein presented show that Lut7 display high antioxidant capacity. This potential is especially relevant since neuronal cells present a high metabolic rate, continuously generating reactive oxygen species (ROS) during aerobic metabolism, as a result of electron transport chain (ETC) action during oxidative phosphorylation. As a consequence, brain tissue is particularly susceptible to oxidative stress [39,40]. Several events have been associated with neurodegeneration such as synaptic dysfunction, excitotoxicity, and oxidative stress. Indeed, because of its high metabolic rate combined with a limited capacity of cellular regeneration, the brain is particularly sensitive to oxidative damage. 

The neurotoxin 6-OHDA is a potent inhibitor of complex I and causes direct oxidative damage through superoxide and hydrogen peroxide production and indirect damage after suffering auto-oxidation, generating even more ROS [41,42,43,44]. In order to evaluate ROS production and the capacity of Lut7 to promote cell recovery from 6-OHDA-induced damage, undifferentiated SH-SY5Y cells were used. Our findings revealed that Lut7 did not affect ROS production. Another study reported that Lut7 did not interfere on hepatitis B virus-induced intracellular ROS accumulation in HepG2 cells [45]. However, in contrast to our results, previous studies reported that Lut7 decreases ROS in many cell lines or in vivo models. Palombo et al. evaluated ROS production in IL-22 or IL-6-stimulated human keratinocytes (HEKn cells) and verified that at 20 µM, Lut7 treatment reduced ROS generation [46]. Similarly, in HUVEC cells (human umbilical vein endothelial cells) Lut7 at 20 µM reduced ROS generation and downregulated genes involved in inflammation [47]. The divergent results found in literature may derive from different cellular responses to different Lut7 concentrations, and/or different cell lines. 

A correlation between mitochondrial membrane potential (ΔΨm) and reactive oxygen species (ROS) production has been demonstrated [48,49,50]. Additionally, Δψm depolarisation is usually correlated with neuronal death [51,52]. In this case, the present results indicate that Lut7 reverted the 6-OHDA-induced Δψm depolarisation but it was not able to reduce the ROS production [45]. The MMP recovery mediated by Lut7 observed in SH-SY5Y cells after 6-OHDA-induced cell injury was similar to a previous study conducted with cisplatin in HK-2 cells (human proximal tubule cell line). According to Nho et al., (2018) Lut7 decreased cell death, promoted a recovery in MMP and abolished Caspase-3 activity [53]. In this study, Lut7 also decreased Caspase-3 activity in SH-SY5Y cells exposed to 6-OHDA. In H9c2 cells (rat cardiomyoblast cell line), Lut7 pretreatment reduced apoptosis, intracellular ROS, chromatin condensation and DNA damage, and reverted mitochondrial dysfunction induced by doxorubicin [54]. Another study reported similar results in H9c2 cells (reduction of apoptosis, ROS generation and mitochondrial dysfunction) but also showed downregulation of Caspase-3, p-ERK1/2, p-JNK and p-P38 inhibition, and p-ERK5 activation in angiotensin II-induced cells [55].

It was reported that mitochondrial dysfunction is mediated by JNK activation, while its inhibition prevent both the loss of Δψm and apoptotic events [56,57,58,59]. One possible explanation for these findings is that JNK plays a significant role in apoptosis via the intrinsic pathway (also known as the ‘mitochondrial pathway’), which is activated by extracellular or intracellular perturbations usually found in AD, such as oxidative stress. In response to a deleterious stimulus (such as ROS), JNK phosphorylates 14-3-3 protein, and induces the translocation of pro-apoptotic proteins (Bax and Bad) from the cytoplasm to the mitochondria, the major source of ROS in cells. The translocation of these pro-apoptotic proteins induces mitochondrial outer membrane permeabilization (MOMP), allowing the cytosolic release of pro-apoptogenic factors that normally reside in the mitochondrial intermembrane space, such as cytochrome c and Smac/DIABLO [60,61]. Cytochrome c then associates with Apaf-1, pro-Caspase 9 (CASP9), (and possibly other proteins) to form an apoptosome, which activates CASP9. When activated, CASP9 catalyzes the proteolytic activation of CASP3 and CASP7 (known as ‘executioner caspases’), which handle cell demolition during intrinsic and extrinsic apoptosis pathways. However, DNA damage can also activate JNK. p53 is another JNK substrate that induces expression of pro-apoptotic genes (puma, fas and bax), leading to apoptosis in a mitochondrial-independent manner. On the other hand, p53 can trigger the MOMP as well in a transcription-independent manner by activating pro-apoptotic Bcl-2 proteins (Bax or Bak) or by inactivating anti-apoptotic Bcl-2 proteins (Bcl-2 and Bcl-X1) [62,63]. 

Recently, the role of JNK3 in Alzheimer’s disease (AD) was reviewed [64]. It was demonstrated that synthetic JNK3 inhibitors have a promising future as therapeutic alternatives for AD treatment as they appear to attenuate many neurodegenerative-associated phenomena in different models. Interestingly, Lut7 also showed important JNK3 selectivity. The IC_50_ for JNK3 was reported to be as low as 2.45 ± 0.1 µM, while the IC_50_ for p38α was 87.1 ± 2.1 µM, indicating a 35-fold increase of selectivity to JNK3 over p38. Authors hypothesized that the selectivity for JNK3 is a result of the interaction of Lut7 and the residues Asn152, Gln155, Asn 194 and Ser 193 of JNK3 [6]. Therefore, according to our results, it is possible that the main mechanism of Lut7 in preventing mitochondrial-dependent apoptosis may be related with JNK3 inhibition. 

Although many drugs have been evaluated as AD treatment in vivo, in vitro and in silico models, one of the best pharmacological alternatives by far, still consist in AChE inhibitors (AChEI), such as galantamine, rivastigmine or donepezil. It is believed that in early or mid-stages of AD, the increase of ACh induced by AChEI compensates the loss of cholinergic neurons and prolongs the cholinergic sinalization, improving cognitive symptoms, such as learning and memory impairment [65,66,67]. Indeed, AChEIs have been used for over 30 years to increase the levels of ACh in muscarinic and nicotinic receptors, and interestingly, galantamine, an AChEI which has been approved since 2001 by the FDA in the treatment of AD, was first isolated from botanical sources back in the 1950s [68], which reinforces the potential of natural products in providing efficient treatments to several diseases.

Here, we demonstrated that Lut7 is able to inhibit AChE activity in 6-OHDA-treated cells. Corroborating with our results, the role of flavonoids (including Lut7) as potential AChEI have been reviewed elsewhere [13,69,70]. The AChE inhibitory capacity of Lut7 has been also demonstrated by in vivo and in silico studies [71,72,73] despite other studies having reported a wide range of IC_50_ values, probably due to distinct methodologies. The in vitro study conducted by Liu et al. (2020) indicated an IC_50_ of 18.24 ± 2.33 μM [73], while the in vitro and in silico study of Sevindik et al. (2015) identified an IC_50_ of 1.65 μM to AChEI [72]. 

Both toxic protein accumulation and oxidative stress are main hallmarks of NDs and contribute to neuroinflammation, further worsening the disease. Previously, studies had already reported that luteolin suppressed the production of proinflammatory cytokines in macrophages by blocking kappa B (NF-kB), and activator protein 1 (AP1) nuclear signaling pathways, and inhibited the production of nitric oxide and proinflammatory eicosanoids. In addition, luteolin decreased the release of TNF-α and superoxide after LPS induced in microglial cell cultures, and reduced the production of LPS-induced IL-6 in cerebral microglia in vivo and in vitro [74,75]. In the CNS, it decreased inflammation and axonal damage by preventing monocyte migration through the blood–brain barrier (BBB) [3,4,5]. Since both microglia cells and RAW264.7 cells are capable of expressing major histocompatibility complex (MHC) antigens, as well as T and B cell markers and share other phenotypic traits and innate immunological functions with other mononuclear phagocytes, we also demonstrated that Lut7 can reduce TNF-α after 3 h, and increase IL-10 after 24 h. In the past two decades, neuroinflammation has been considered an important component of the NDs’ pathogenesis. It is well established that the CNS is composed of distinct kinds of cells that perform specific roles in brain homeostasis and therefore, may contribute differently to the worsening of symptoms or progression of ND.

A previous study reported that pretreatment with Lut7 suppressed the induction of nitrite, ROS, PGE2, and TNF-α in a dose-dependent manner in IL-1β-stimulated rat primary chondrocytes [76], suggesting that Lut7 has a potent anti-inflammatory effect. Additionally, Lut7 inhibited the IL-1β-induced nuclear accumulation of NF-κB subunit p65 by suppressing phosphorylation and degradation of IκB-α and significantly inhibited the IL-1β-induced phosphorylation of ERK, JNK, and p38 MAPK in a dose-dependent manner [76].

Despite the promising potential of Lut7, there are some limitations in our study that should be addressed in future research. The primary focus of our study was to evaluate the neuroprotective, antioxidant and anti-inflammatory effect of Lut7 in in vitro models of neurodegenerative diseases, yet, further studies are necessary to attest the results here described. Therefore, evaluation of inflammatory mediators such as iNOS and COX-2, as well as the expression of transcription factors (e.g., Nrf2, AP1, NF-kB), antioxidant enzymes (e.g., SOD, GSH-Px), and other apoptosis-related proteins (e.g., Caspase-9 and -8, Bax, cytochrome C, JNK, p38) would be of extreme value to fully elucidate the anti-inflammatory, anti-apoptotic and antioxidant mechanisms of Lut7, especially based on co-culturing systems (e.g., neuron and microglia-derived cells co-culture, 3D) or more complex models (e.g., in vivo models) of neurodegenerative diseases.

## 5. Conclusions

**Figure 6:**
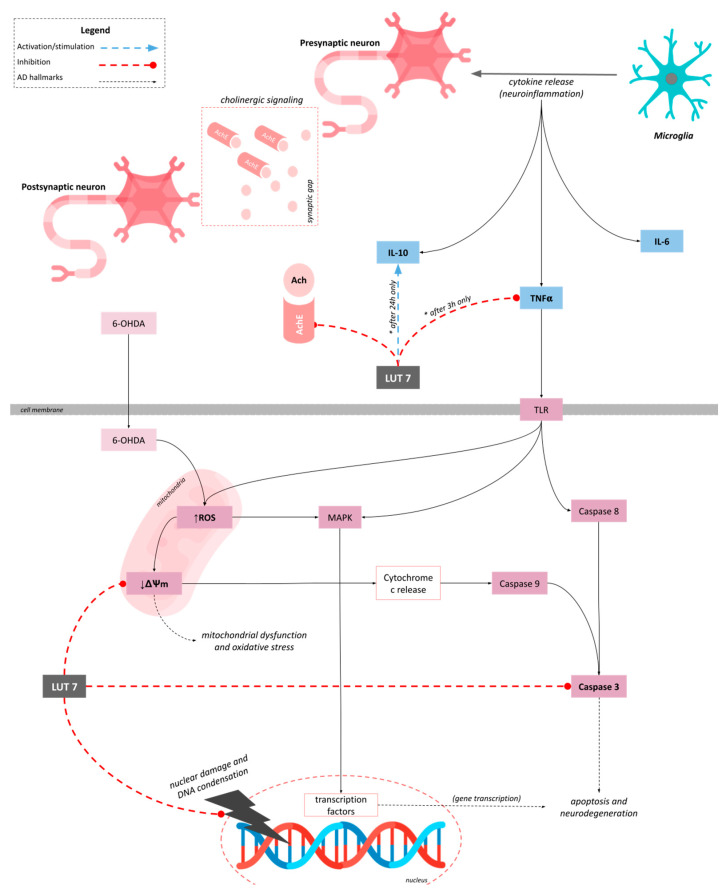
Proposed mechanism of action of Luteolin-7-O-glucoside (Lut7). Regarding the mitochondria, Lut7 prevented membrane depolarization induced by 6-OHDA and indirectly reduced mitochondrial dysfunction and oxidative stress. Lut7 also decreased Caspase-3 activity protecting cells against 6-OHDA-induced apoptosis. Additionally, at low concentrations, Lut7 was able to inhibit AChE activity, which may contribute to alleviating AD symptoms. In RAW264.7 cells, Lut7 was able to reduce TNF-α production (after 3 h), and induce IL-10 release (after 24 h), which may contribute to modulate the neuroinflammation.

In summary, our results suggested that Lut7 protected SH-SY5Y cell line against 6-OHDA-induced damage and protected differentiated SH-SY5Y cells against neurotoxicity induced by 6-OHDA over 48 h. Although we did not observe a reduction of ROS production, Lut7 protected SH-SY5Y cells against 6-OHDA-induced mitochondrial and nuclear damage and reduced Caspase-3 and AChE activity. In RAW264.7 cell line, Lut7 was able to decrease TNF-α and to increase IL-10 levels (Figure 6). 

Several questions remain unanswered but we believe this is an interesting topic for future work and may be a good starting point for further investigation in in vivo models of neurodegenerative diseases to validate Lut7 pharmacological potential.

## Figures and Tables

**Table 1:** Antioxidant activity of Lut7 and BHT.

	DPPH ^(A)^	FRAP ^(B)^	ORAC ^(C)^
Lut7	6.8 (0.76–0.9)	19,570.78 ± 291.48	8804.19 ± 409.99
BHT	>100	2821.50 ± 63.03	143.70 ± 23.36

BHT (butylated hydroxytoluene) was used as a standard. The values in the table represent the mean ± SEM from 3 independent experiments. ^(A)^ radical scavenging activity (EC_50_ µg/mL); ^(B)^ µM of FeSO_4_ per gram of compound; ^(C)^ µM of Trolox equivalent (TE)/g of compound.

## Data Availability

The data presented in this study are available on request from the corresponding author.
